# Cardiometabolic Comorbidity Risk in Pediatric Patients With Psychiatric Illnesses: A Case-Control Inpatient Study

**DOI:** 10.7759/cureus.26326

**Published:** 2022-06-25

**Authors:** Hadeel Dweik, Jaskaranpreet Kaur, Sanobar Jaka, Farzana Faruki, Rushi P Shah, Ozge C Amuk Williams, Ankit Chalia, Anil Bachu

**Affiliations:** 1 Medicine, The University of Jordan, Amman, JOR; 2 Internal Medicine, North Alabama Medical Center, Florence, USA; 3 School of Global Public Health, New York University, New York, USA; 4 Psychiatry, Essen Health Care, Bronx, USA; 5 Medicine, Byramjee Jeejeebhoy Medical College, Ahmedabad, IND; 6 Psychiatry, Griffin Memorial Hospital, Norman, USA; 7 Behavioral Medicine & Psychiatry, West Virginia University School of Medicine, Martinsburg, USA; 8 Psychiatry, University of Arkansas for Medical Sciences, North Little Rock, USA

**Keywords:** risk-factors, cardiometabolic conditions, child and adolescent, obesity and diabetes, psychiatric comorbidities

## Abstract

Objectives

To delineate the differences in the cardiometabolic comorbidities in pediatric patients with medical versus psychiatric illnesses and to determine the risk of association between the spectrum of cardiometabolic comorbidities in pediatric patients with a broad range of psychiatric illnesses.

Methods

We conducted a case-control study using the nationwide inpatient sample (NIS), the largest hospital database in the United States (US) and included 179,550 pediatric patients (age 10-18 years) that were hospitalized with a primary diagnosis of psychiatric illness (N = 89,775) and pediatric patients that were hospitalized with a primary diagnosis of medical illness (N = 89,775). We used descriptive statistics and Pearson’s chi-square test to delineate the differences between pediatric inpatients with medical versus psychiatric illnesses.

Results

The majority of pediatric patients with psychiatric illnesses were females (58%) and white (62%), with a mean age of 15 years. Cardiometabolic comorbidities were higher in patients admitted for psychiatric illness, with a higher prevalence of hypothyroidism (1.6%) and obesity (7.1%) than in those hospitalized for medical illnesses. Among all cardiometabolic comorbidities, obesity had the highest prevalence across all psychiatric illnesses, measuring eight percent in patients with disruptive behavior disorders, followed by seven percent each in anxiety, mood, and psychotic disorders. Diabetes had the lowest prevalence hovering between one and two percent for a spectrum of psychiatric illnesses.

Conclusion

The prevalence of cardiometabolic comorbidities is higher in pediatric inpatients with psychiatric illnesses. This calls for timely monitoring of the routine labs and early diagnosis and management of the cardiometabolic comorbidities in this at-risk population.

## Introduction

The prevalence of cardiometabolic disease in the pediatric population can be difficult to evaluate due to a lack of unified diagnostic methodology [1]. This is further complicated by varying definitions and diagnostic criteria for metabolic syndrome in pediatric patients that focus primarily on waist circumference as a surrogate measure of central obesity [2]. Despite these challenges with measurement, the current observed trend of rising childhood cardiometabolic morbidity has been attributed to a rise in obesity rates [3]. A study on the National Health and Nutrition Examination Survey found that nearly 75% of its adolescent participants had at least one metabolic pathology and about one in ten adolescents had metabolic syndrome [4].

Interestingly, studies have also found an increased prevalence of cardiometabolic comorbidities in patients with psychiatric illness due to an increased likelihood of developing risk factors such as dyslipidemia, hypertension, and hyperglycemia [5]. These cardiometabolic risk factors are also independently associated with commonly used psychotropic medications, particularly antipsychotics, mood stabilizers, and a few antidepressants adding to the risk of developing cardiometabolic risk factors [6].

In the US, of all the children and adolescents diagnosed with diabetes in the 1990s, only three percent of those diagnoses accounted for type-II diabetes [7]. However, in the 2010s, type-II diabetes accounted for nearly 30% of diabetes in the pediatric population [8]. Eighty percent of obese children with at least one cardiometabolic risk factor experienced limitations in their quality of life (QoL) as noted by the patients and their parents [9]. The pediatric cardiometabolic syndrome is alarming as it may persist in adulthood and increase the future risk of diabetes and cardiovascular disease [1]. The lack of current literature on the potential association between psychiatric illnesses and cardiometabolic comorbidities in this population prompts our national inpatient database study to investigate this potential area of research. Therefore, we conducted a case-control study to delineate the differences in the cardiometabolic comorbidities in pediatric patients with primary medical diagnosis versus primary psychiatric diagnosis and to determine the prevalence of these cardiometabolic comorbidities in pediatric patients with a broad range of psychiatric illnesses.

## Materials and methods

Study sample

We conducted a case-control study using the nationwide inpatient sample (NIS), the largest hospital database in the United States (US) and included 179,550 pediatric patients (age 10-18 years) that were hospitalized with a primary diagnosis of psychiatric illness (N = 89,775) and pediatric patients that were hospitalized with a primary diagnosis of medical illness (N = 89,775) [10]. The group of inpatients with medical illness was obtained by propensity case-control age-matching and a match tolerance set at zero.

Variables

We included demographic characteristics (age, sex, and race) and coexisting relevant diagnoses in the inpatient record. The cardiometabolic comorbidities included hypothyroidism, diabetes, hypertension, and obesity. The primary psychiatric illnesses of pediatric patients were anxiety disorders, disruptive behavior disorders (DBDs), mood disorders, psychotic disorders, and substance use disorders (SUDs).

Statistical analysis

We used descriptive statistics and Pearson’s chi-square test to delineate the differences between pediatric inpatients with medical versus psychiatric illnesses. All analyses were conducted using the Statistical Package for the Social Sciences Version 26.0 (IBM Corp., Armonk, NY), and statistical significance was set to a two-sided P-value of <0.05.

Ethical approval

The NIS is publicly available de-identified data with the protection of patients, physicians, and hospital-related information. This study did not require approval from the institutional review board.

## Results

Among pediatric inpatients, both psychiatric and medical illnesses were more prevalent in females and whites. Compared to the medical inpatients, there was a statistically significant higher prevalence of psychiatric illness in the white patients (61.8% vs. 55.7%), compared to black and Hispanic patients. Among cardiometabolic comorbidities, the prevalence of obesity (7.1% vs. 3.9%) and hypothyroidism (1.6% vs. 1.2%) was higher in psychiatric inpatients compared to the medical inpatients. Comorbid diabetes and hypertension were prevalent among medical inpatients as shown in Table 1.

**Table 1:** Distribution of pediatric inpatients by illness type. SD: standard deviation.

Variable	Medical illness	Psychiatric illness	P value
Mean age (SD)	15.0 (2.16)	15.0 (2.16)	1.00
Sex, in %			
Male	41.3	42.0	
Female	58.7	58.0	0.002
Race, in %			
White	55.7	61.8	<0.001
Black	20.9	17.8	
Hispanic	15.8	12.9	
Other	7.6	7.5	
Cardiometabolic comorbidities, in %			
Hypothyroidism	1.2	1.6	<0.001
Diabetes	1.6	1.4	0.013
Hypertension	2.7	1.3	<0.001
Obesity	3.9	7.1	<0.001

Among all cardiometabolic comorbidities, obesity had the highest prevalence across all psychiatric illnesses, measuring eight percent in patients with DBD, followed by seven percent each in anxiety, mood, and psychotic disorders. Diabetes had the lowest prevalence hovering between one and two percent for a spectrum of psychiatric illnesses. Furthermore, patients with SUD were the least likely to develop cardiometabolic comorbidities, except for hypertension, which was more prevalent compared to other psychiatric illnesses. The prevalence of each cardiometabolic comorbidity was similar across all fields with the exception of obesity, which was found to be at four percent in medical illness but seven to eight percent for four out of five psychiatric illnesses as shown in Table 2.

**Table 2:** Cardiometabolic comorbidities by illnesses.

Comorbidity	Medical illness	Anxiety disorders	Disruptive behavior disorder	Mood disorders	Psychotic disorders	Substance use disorder
Hypothyroidism	1%	1%	2%	2%	2%	1%
Diabetes	2%	1%	1%	2%	1%	1%
Hypertension	3%	1%	1%	1%	3%	2%
Obesity	4%	7%	8%	7%	7%	2%

## Discussion

The most notable finding of our study demonstrates an increased prevalence of obesity in pediatric psychiatric inpatients compared to pediatric medical inpatients. It emphasizes obesity as the most common cardiometabolic comorbidity in pediatric psychiatric inpatients. The mechanism of obesity in psychiatric illnesses is thought to be a multifactorial model. A lifestyle component, as implicated by O’Neil et al., may lead to a higher intake of processed foods as well as overeating and binge-eating habits [11,12]. Decreased physical activity in psychiatric patients with depression was another lifestyle factor found to contribute to obesity [13]. Antipsychotic medications, especially second-generation antipsychotics like risperidone, represent another contributing factor [14]. Although antipsychotic-induced weight gain is seen in schizophrenic patients who have discontinued their antipsychotic medications, however, there has been no clear explanation of this finding in the literature [15]. In addition, genetic and biological factors, such as shared genes between obesity and mental disorders, may contribute to the prevalence of obesity in psychiatric patients. For example, 12% of the genes that make up for depression also represent genes that lead to obesity [16]. Leptin insufficiency and leptin resistance in depressed patients have been implicated as mediators of obesity in these patients, as do disruption of the circadian rhythm and neurotransmitter dysregulation [17-19].

Data on the prevalence of type-II diabetes in the pediatric population shows a clear rise, as discussed in the introduction. When accounting for the prevalence of pediatric hypertension, current data reveals that one to five percent of youth people is diagnosed with hypertension [20,21]. A meta-analysis by Ogden et al. found a staggering increase of 75-79% in the prevalence of hypertension in children between 2000 and 2015. In terms of obesity prevalence, about 22.8% of preschool children, 34.2% of six to 11-year-old, and 34.5% of adolescents 12 to 19 years of age were found to be overweight and obese [22]. This rapid rise and high prevalence of cardiometabolic disease, especially in a population vulnerable to its long-term effects, warrant exploration of additional, previously unidentified risk factors that can aid in risk mitigation, diagnosis, and early detection of disease prevention and modification. Of the many etiologies and risk factors that have been identified in the pathophysiology of pediatric diabetes and hypertension, psychiatric illness has never been one of them. Even though there have been enormous data to support an increased incidence of diabetes and hypertension in obese patients, these studies do not specifically highlight obesity’s association with primary psychiatric illness [23-25].

In addition, chronic kidney disease, Cushing syndrome, and coarctation of the aorta have all been well established as causes of pediatric hypertension. There are no studies looking at associations with pediatric psychiatric illnesses [26,27]. There seems to be scarce data on the prevalence of diabetes and hypertension in pediatric patients with psychiatric disorders. Therefore, no comparison can be made regarding the prevalence of these comorbidities in pediatric psychiatric inpatients and pediatric medical inpatients.

This is a cross-sectional study and therefore, as a limitation of this study type, we cannot establish a causal relationship between cardiometabolic comorbidities and psychiatric disorders. It is not possible to point out if the medical illness occurred before the psychiatric illness or vice versa. We have studied the prevalence of comorbidities by common psychiatric illnesses. However, it was not possible to divide and study medical illnesses in a similar manner. Our focus and goal were to compare the prevalence of cardiometabolic comorbidities in hospitalized patients with a primary medical diagnosis and hospitalized patients with a primary psychiatric diagnosis. Also, the NIS dataset may under-report other comorbidities that may or may not be pre-existing conditions or other diagnoses in the patient records, which can potentially act as a confounding factor. Despite these limitations, the NIS offers a large database that provides a national presentation of the population with results that are generalizable to the inpatient population.

## Conclusions

Pediatric patients with psychiatric illnesses are among the most vulnerable patient populations. Our findings identify a higher prevalence of obesity among primary psychiatric illnesses than one with primary medical illnesses. The dramatic rise in childhood cardiometabolic comorbidities, warrants closer monitoring of metabolic panel labs, early diagnosis and management of cardiometabolic comorbidities in this at-risk population. Identification of psychiatric illness in a clinical setting should warrant a concomitant focus on lifestyle modification, nutritional counseling, and caregiver education. An integrated approach towards medical and psychiatric illness management will allow for the mitigation of long-term medical complications from obesity, improved QoL, and lower health care costs.
